# Comfort and Adverse Reactions in Immediate Mouthwash Use: A Comparative Study

**DOI:** 10.3290/j.ohpd.c_2856

**Published:** 2026-11-06

**Authors:** Priscila Ladeira Casado, Bruna Ghiraldini, Fabio Jose Barbosa Bezerra, Sarah Pimentel, Fabricio da Silva Pereira, Larissa Moreira de Souza, Luiza dos Anjos Motta, Alexandre Montenegro

**Affiliations:** a Priscila Ladeira Casado Professor, School of Dentistry, Universidade Federal Fluminense, Niterói, RJ, Brazil. Experimental design, performed the experiments, proofread the manuscript, consulted on and performed statistical evaluation, contributed substantially to discussion, draft preparation; writing – review and editing.; b Bruna Ghiraldini Researcher, Universidade Paulista, São Paulo, SP, Brazil. Experimental design, performed the experiments, proofread the manuscript, consulted on and performed statistical evaluation, contributed substantially to discussion, draft preparation; writing – review and editing.; c Fabio Jose Barbosa Bezerra Professor, School of Dentistry, Universidade de São Paulo, São Paulo, SP, Brazil. Idea, experimental design, conceptualisation, proofread the manuscript, consulted on and performed statistical evaluation, contributed substantially to discussion, draft preparation; writing – review and editing.; d Sarah Pimentel Graduate Student, School of Dentistry, Universidade Federal Fluminense, Niterói, RJ, Brazil. Conducted the experiments, performed clinical evaluations, recruited participants, carried out statistical analysis, and documented photographs.; e Fabricio da Silva Pereira Graduate Student, School of Dentistry, Universidade Federal Fluminense, Niterói, RJ, Brazil. Conducted the experiments, performed clinical evaluations, recruited participants, carried out statistical analysis, and documented photographs.; f Larissa Moreira de Souza MSc in Dentistry, School of Dentistry, Universidade Federal Fluminense, Niterói, RJ, Brazil. Conducted the experiments, performed clinical evaluations, recruited participants, carried out statistical analysis, and documented photographs; draft preparation; writing – review.; g Luiza dos Anjos Motta PhD, School of Dentistry, Universidade Federal Fluminense, Niterói, RJ, Brazil. Conducted the experiments, performed clinical evaluations, recruited participants, carried out statistical analysis, and documented photographs, draft preparation; writing – review.; h Alexandre Montenegro DDS, MsC, PhD Brazilian Navy, Coordinator of the Brazilian Dental Clinic. Reasearcher, School of Dentistry, Universidade Federal Fluminense, Niterói, RJ, Brazil.

**Keywords:** chlorhexidine, essential oils, green tea, hyaluronic acid, mouthwash

## Abstract

**Purpose:**

To evaluate the immediate effects and user acceptance of mouthwashes containing different active ingredients – chlorhexidine, essential oils, and green tea/hyaluronic acid – by assessing their impact on salivary pH, sensory experiences, and potential side effects.

**Methods and Materials:**

This is a randomised clinical trial, including 96 students who were randomised into three groups, each using one of three mouthwashes: essential oils, chlorhexidine, and green tea/hyaluronic acid. Participants conducted a 60-s rinse and subsequently filled out questionnaires on sensory experiences, such as taste and irritation, and the potential for daily use. Salivary pH was measured immediately before and after rinsing to assess any changes.

**Results:**

All mouthwashes effectively increased salivary pH from an average of 7.0 to 8.6, indicating a shift towards a less acidic oral environment. There were no significant differences in the sensation of clean teeth across different mouthwashes. However, the green tea/hyaluronic acid mouthwash was more favourably received, causing less discomfort in the lips, tongue, and throat and showing no reports of tongue irritation. Participants expressed a significantly higher preference for this mouthwash (*p* = 0.04) for potential daily use, as opposed to those using essential oil or chlorhexidine mouthwashes, which commonly reported burning sensations.

**Conclusion:**

Although all tested mouthwashes were effective in increasing salivary pH, the green tea/hyaluronic acid formulation stood out due to higher user comfort and acceptance, potentially due to its natural component base. These findings suggest a promising direction for future mouthwash formulations that prioritise natural ingredients and user comfort. In conclusion, green tea and hyaluronic acid mouthwash increases salivary pH with better user comfort and acceptance than chlorhexidine or essential oils.

Oral health is an integral component of overall health, directly influencing the quality of life and well-being of individuals of all ages. Inadequate oral hygiene can lead to various complications, including cavities and periodontal diseases.^3^ Periodontopathogenic bacteria are not only instrumental in oral infections but also significantly contribute to systemic conditions such as cardiovascular disease, Alzheimer’s disease^7^ and diabetes, underscoring the critical link between oral and overall health.^13^ While tooth brushing and flossing are primary methods for maintaining oral hygiene, mouthwashes have proven to be an effective complementary tool, containing antimicrobial agents capable of reducing plaque formation, helping combat bad breath (halitosis), and, in some cases, delivering therapeutic agents.^4,10–13,19^


In recent years, there has been a growing interest in the use of natural products and compounds with multiple bioactive properties in the development of mouthwashes. Notable formulations in this regard incorporate green tea extracts, tea tree oil, hyaluronic acid, DSBC (dilaneidol salicylate),^4,17
^ essential oils,^21^ and chlorhexidine-based active ingredients.^5^


One of the primary mouthwashes used in dental practice, chlorhexidine, is considered highly effective against a range of oral microorganisms. Chlorhexidine mainly functions by disrupting the bacterial cell membrane, increasing its permeability, and consequently promoting the leakage of cellular contents, leading to the microorganism’s death. This mode of action is valid for both Gram-positive and Gram-negative bacteria, as well as some types of fungi and viruses. Due to its broad efficacy, chlorhexidine is often referred to as a broad-spectrum antimicrobial agent, reducing the microbial load in the oral cavity.^14,15,26
^ However, one of the most common side effects of prolonged chlorhexidine use is tooth and dental restoration staining. This occurs due to the interaction of chlorhexidine with salivary proteins, resulting in brown or yellow discolouration caused by pellicle formation,^1^ and taste alterations, including a metallic taste. Although this effect is typically temporary, it can be uncomfortable for some patients and may affect the appreciation of food and drink flavours.^5^


Mouthwashes with essential oils are formulated from a combination of natural compounds extracted from plants, known for their antimicrobial, anti-inflammatory, and antioxidant properties. The main essential oils frequently used include: (i) thymol, extracted from thyme, with antimicrobial properties against bacteria and fungi; (ii) eucalyptol, derived from eucalyptus, known for its anti-inflammatory and antibacterial properties; (iii) menthol, obtained from peppermint oil, which provides a refreshing sensation and also has an antimicrobial effect; (iv) methyl salicylate, found in wintergreen oil, known for its anti-inflammatory and analgesic properties. These essential oils, in mouthwash form, function by destabilising the bacterial cell membrane, resulting in cell death by lysis.^21,29
^


Green tea (*Camellia sinensis*) is widely recognised for its antioxidant and antimicrobial properties. Studies have shown that its polyphenols, particularly catechins, help reduce plaque formation and decrease gingival inflammation, contributing to more balanced oral health. Tea tree oil (*Melaleuca alternifolia*), commonly known as tea tree, is famous for its antiseptic and antibacterial actions and is effective in combating various species of pathogenic microorganisms that colonise the oral cavity. Studies confirm its efficacy in treating periodontal diseases and reducing bacterial plaque.^6,22,25
^


Additionally, hyaluronic acid, a natural component of the extracellular matrix, plays a crucial role in tissue healing and regeneration. In the field of dentistry, its use is valued for its moisturising, anti-inflammatory, and cell renewal-promoting properties in the oral mucosa, aiding in lesion recovery and gum health maintenance.^9,23
^ Besides these components, DSBC (dilaneidol salicylate) is an innovative compound with promising antimicrobial and anti-inflammatory actions. Initial studies suggest that its incorporation into mouthwashes may represent a novel approach in combating periodontopathogens, providing an additional layer of protection and treatment.^18^


Another crucial factor in the formulation of mouthwashes is the pH, which plays a significant role in the product’s efficacy, including the prevention of dental erosion, control of oral biofilm formation, formulation stability, acid neutralisation, and patient comfort.^8^ Mouthwashes with low (acidic) pH can contribute to enamel demineralisation, increasing the risk of dental erosion. A neutral to slightly alkaline pH is preferable to avoid demineralisation. The pH can influence the burning sensation and comfort in the mouth. Mouthwashes with very acidic or very alkaline pH can cause discomfort and irritation of the oral mucosa, which may discourage regular use.^28^


In this context, combining different active components in a single mouthwash offers a multifaceted approach to oral health maintenance, providing benefits that go beyond mechanical plaque control and may eliminate short- and long-term side effects while maintaining antimicrobial properties. Our hypothesis is that mouthwashes containing green tea/hyaluronic acid, essential oils, and chlorhexidine can differentially affect oral health outcomes, considering sensations and comfort. Therefore, the aim of this study was to compare chlorhexidine-based, essential oil-based, and green tea/hyaluronic acid-based mouthwashes in terms of patient perception of possible side effects, immediate action on salivary pH, and sensations related to aroma, taste, burning, and oral irritation, and to include information about sustainability.

## Methods and Materials

This randomised, double-blind clinical trial was approved by the Research Ethics Committee of Antônio Pedro University Hospital/Fluminense Federal University, under number 7.314.194 and included a total of 96 research participants, comprising undergraduate and postgraduate students from the School of Dentistry at Fluminense Federal University, Brazil. All participants signed an informed consent form, agreeing to their participation in the study. Inclusion criteria were: age over 18 years; being a student at the UFF School of Dentistry; and having partial or full dentition.

Based on the eligibility criteria, the 96 participants were divided into three groups to perform a rinse with 15 ml of three different commercially available mouthwashes, identified as A, B, and C: Group A (n = 32); Group B (n = 32); Group C (n = 32). The mouthwashes were provided without brand identification, in identical bottles labelled A, B, and C (Fig 1). The characteristics of the mouthwashes are described in Figure 2. The randomisation procedure was conducted by another operator to ensure impartiality. The allocation sequence was generated using the website www.random.org, which provided unrestricted random number generation for participant randomisation.

**Fig 1 Fig1:**
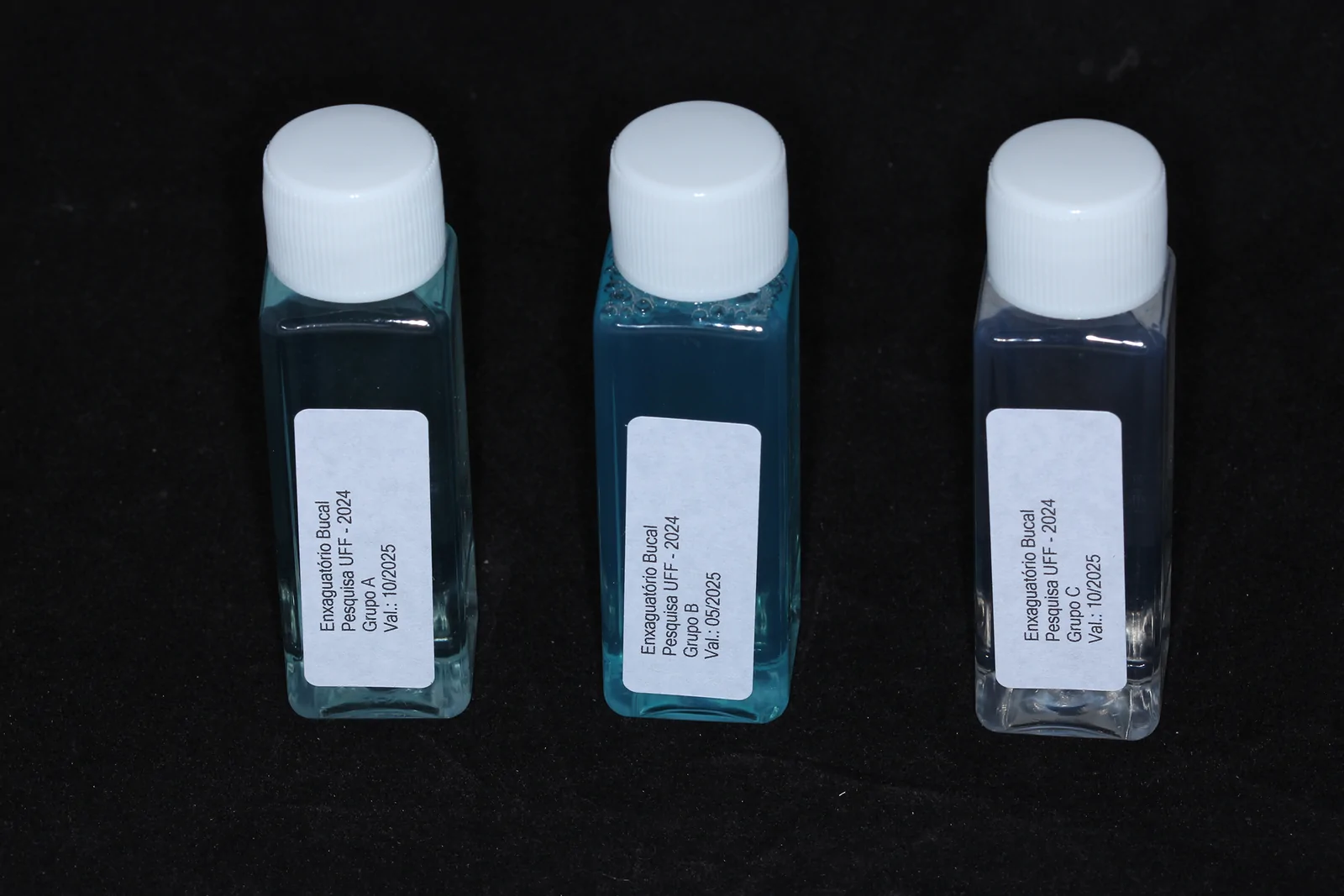
Bottles containing 15 ml of mouthwashes A, B, and C, without commercial identification of the products.

**Fig 2 Fig2:**
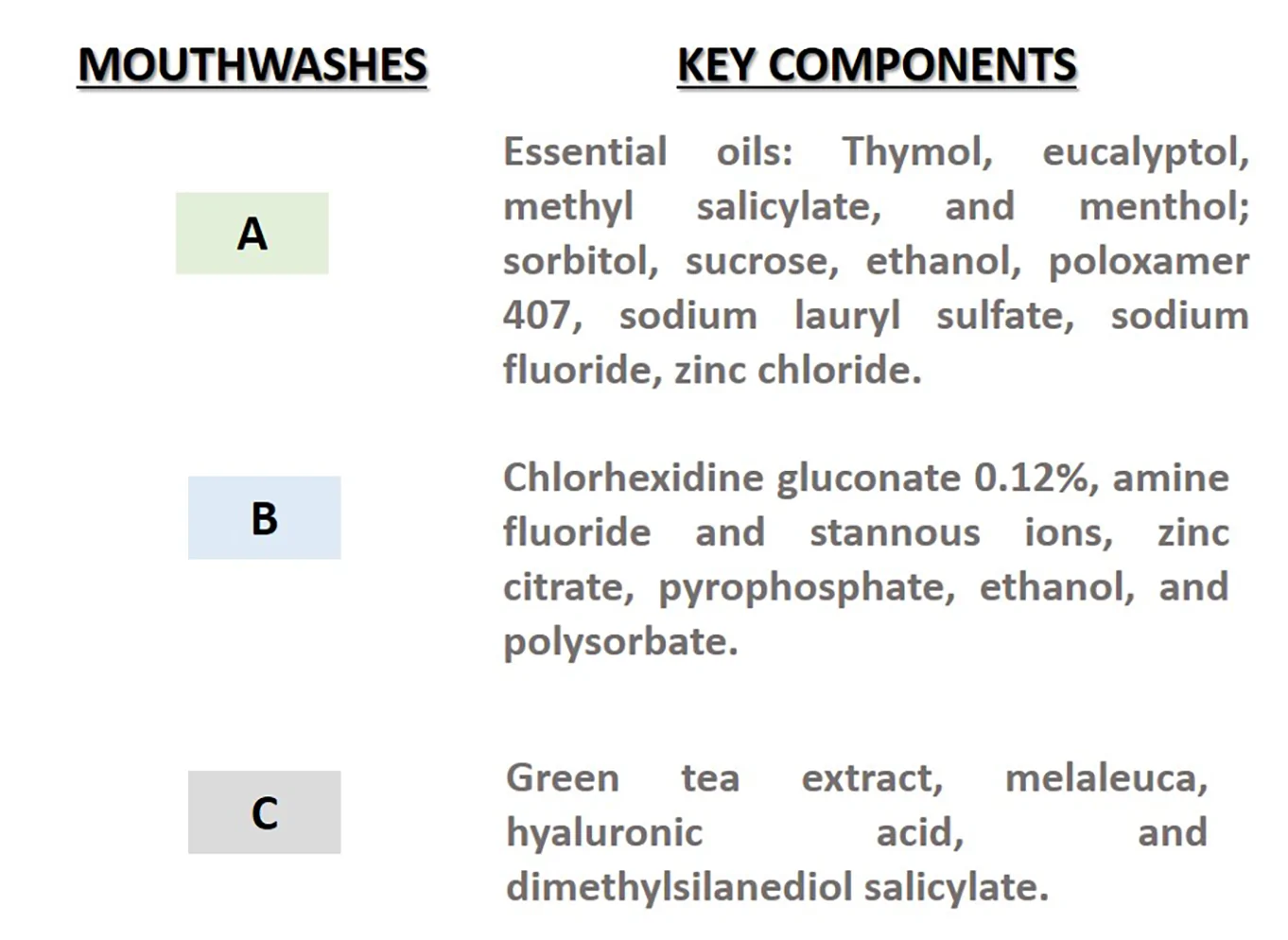
Key chemical components of the mouthwashes used in the study: A, B, and C.

Participants were instructed to rinse with the selected mouthwash for 60 s and subsequently respond to a questionnaire with relevant information regarding the use of the rinse, including sensations of cleanliness, taste, bitterness, any potential discomfort in the oral cavity, irritation, burning sensation, and the potential for daily use.

The measurement of salivary pH (pH 1–14 Test Paper, Labaider, Maharashtra, India) was conducted according to the manufacturer’s instructions by maintaining contact with the saliva for 5 s in the sublingual area, at two different times: immediately before rinsing with the mouthwash and immediately after discarding the mouthwash (Fig 3).

**Fig 3a and b Fig3aandb:**
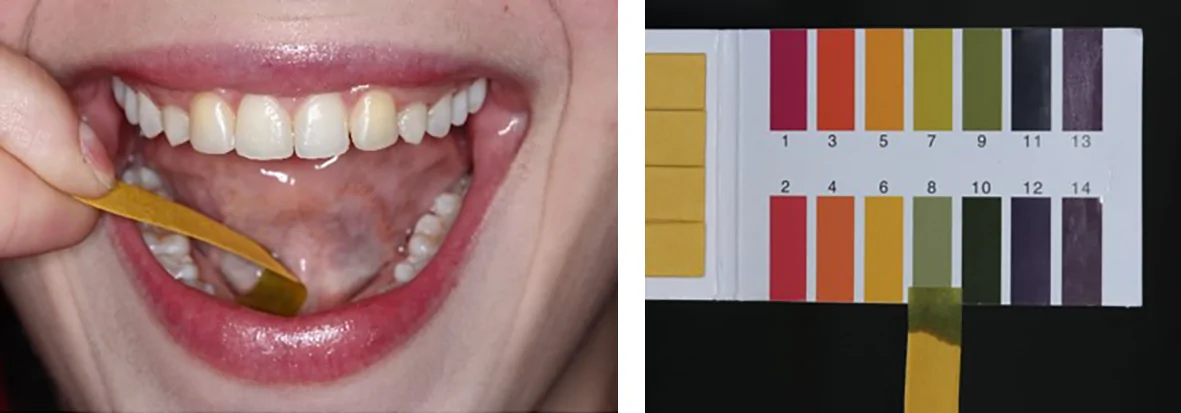
Procedure for salivary pH analysis: (a) Measurement of salivary pH with strips inserted in the sublingual area; (b) Interpretation of the pH value according to the referenced colour standard.

After the completion of rinsing in all groups and statistical analysis of the responses obtained from the questionnaires, groups A, B, and C were identified as mouthwashes from the brands LISTERINE (JNTL Consumer Health, São Paulo, SP – Brazil Ltda) (A), PERIOGARD (Colgate-Palmolive Company®, São Paulo, SP – Brazil) (B), and N&W PRO (New Dental Care Ltda, Ribeirão Preto, SP- Brazil) (C), confirming the double-blind trial. Both the participants and the individuals conducting assessments or interacting with them were unaware of the group assignments until after data collection and analysis were completed.

### Statistical Analysis

The GraphPad Prism 6.0 software (GraphPad Software, La Jolla, CA, USA) was used for all statistical analyses. To address the power of the study, we conducted a sample size calculation based on expected effect sizes and standard deviations from prior studies in similar research. Using a significance level of 0.05 and a power of 80%, the calculated sample size was 96, which is sufficient to detect meaningful differences across the study groups.

Descriptive statistics, including mean, median, and standard deviation, were employed to summarise the data. The analysis of the type of distribution of numerical variables was performed using the Shapiro–Wilk test. The Kruskal–Wallis test was applied to compare differences in the mean values of numerical variables, including age, initial pH, and final pH, among the three groups, due to the non-normal distribution. The Mann–Whitney test was applied to compare numerical variables between groups, as well as within the same group. Pearson’s chi-square test was used to evaluate differences among nominal variables. A *p* value of < 0.05 was considered significant.

## Results

A total of 96 research participants were included in this study, comprising 51 women (53.1%) and 45 men (46.9%), with an average age of 32 ± 12.4 years, with no statistical difference in age among the three groups (*p* = 0.66). The initial average salivary pH before using the mouthwash was 7.0 ± 0.9, and the final average pH after using the mouthwashes was 8.6 ± 0.8, with no statistical difference among the three groups for the initial pH (*p* = 0.51) before rinsing and the final pH (*p* = 0.21) immediately after rinsing.

In the comparison between the initial and final salivary pH within each group, it was evident that there was a significant increase in salivary pH after the use of all the mouthwashes (*p* = 0.00001) (Fig 4).

**Fig 4 Fig4:**
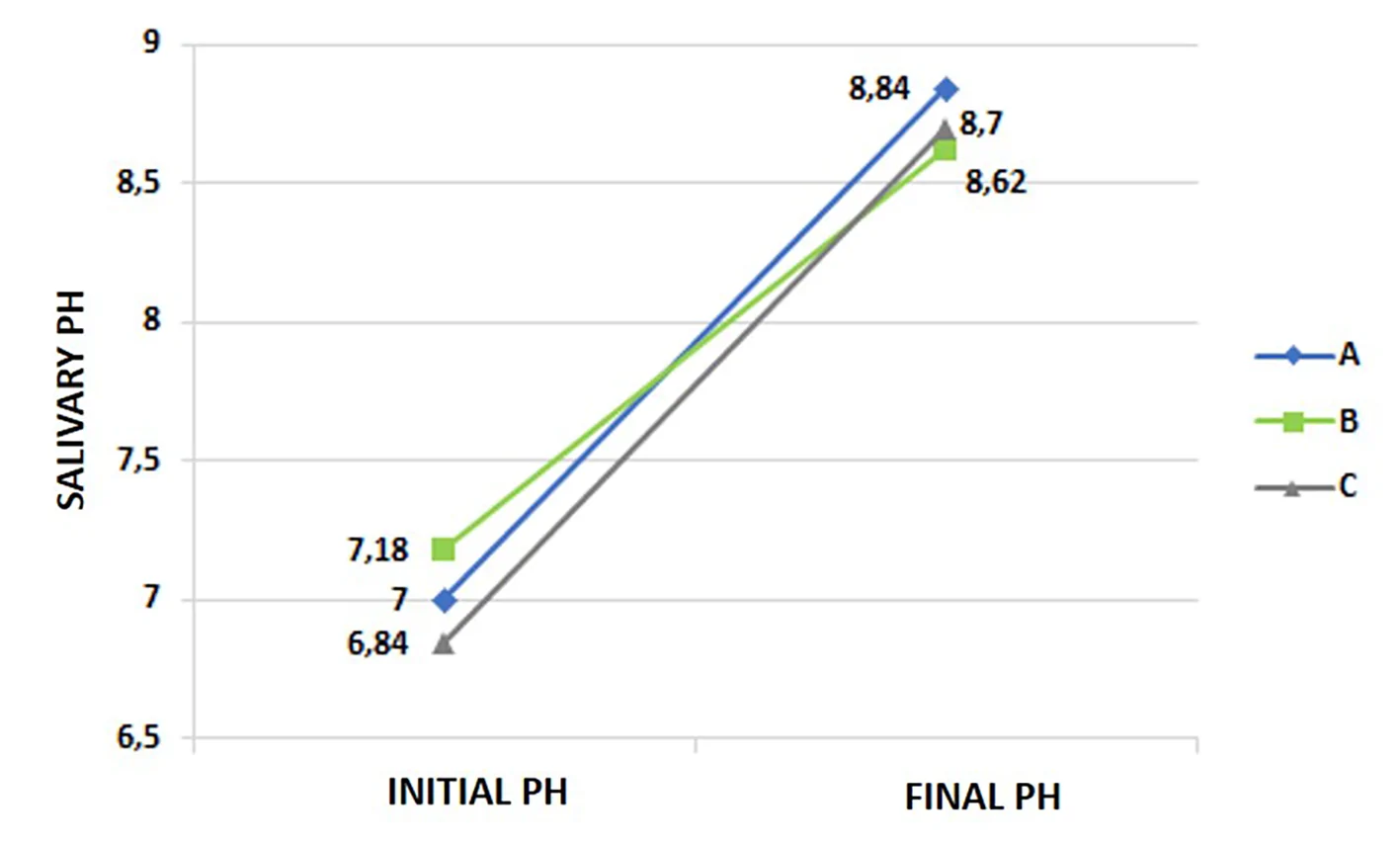
Salivary pH before and after the use of mouthwashes, showing an increase in salivary pH across all three groups (*p* < 0.0001).

Considering the sensory aspects, there was no statistically significant difference between the mouthwashes LISTERINE (A), PERIOGARD (B), and N&W PRO (C) in terms of the sensation of clean teeth, pleasant taste, potential for daily use, and intensity of bitterness, with all three mouthwashes being deemed acceptable for daily use (Table 1).

**Table 1 Table1:** Results highlighting the sensory responses to the administered questionnaire

Participants and sensory analysis	Oral mouthwashes			*p* value
	A (n = 32)	B (n = 32)	C (n = 32)	
Age (years)	30.8 ± 10.8	32.2 ± 12.2	33.9 ± 14.0	0.66
Gender				0.84
Female	16 (50.0%)	18 (56.3%)	17 (53.1%%)	
Male	16 (50.0%)	14 (43.7%)	15 (46.9%%)	
Initial pH	7.0 ± 0.95	7.2 ± 0.93	6.9 ± 0.8	0.51
Final pH	8.8 ± 0.63	8.6 ± 0.75	8.7 ± 0.65	0.21
Did you feel your teeth were clean after use?				0.31
Yes	26 (81.2%)	30 (93.8%)	28 (87.5%)	
No	6 (18.8%)	2 (6.2%)	4 (12.5%)	
Is the taste pleasant?				0.35
Yes	30 (93.8%)	30 (93.8%)	32 (100%)	
No	2 (6.2%)	2 (6.2%)	0	
Would you use the product daily?				0.15
Yes	25 (78.1%)	18 (56.2%)	23 (71.9%)	
No	7 (21.9%)	14 (43.8%)	9 (28.1%)	
Would you buy the product?				0.04
Yes	26 (81.2%)	21 (65.6%)	29 (90.6%)	
No	6 (18.8%)	11 (34.4%)	3 (9.4%)	
What was the intensity of the bitterness?				0.38
As strong as possible	0	1	0	
Very strong	3	3	0	
Strong	4	4	1	
Moderate	5	5	4	
Weak	9	12	17	
Hardly perceived	11	7	10	
Was this intensity acceptable?				0.35
Yes	30 (93.8%)	30 (93.8%)	32 (100%)	
No	2 (6.2%)	2 (6.2%)	0	
Did you feel any discomfort on the lip, tongue, or throat?				0.009
Yes	9 (28.1%)	3 (9.4%)	1 (3.1%)	
No	23 (71.9%)	29 (90.6%)	31 (96.9%)	
Did you feel any burning sensation during use?				0.00009
Yes	20 (62.5%)	9 (28.1%)	4 (12.5%)	
No	12 (37.5%)	23 (71.9%)	28 (87.5%)	
Did you feel any irritation on the tongue during use?				0.03
Yes	6 (18.8%)	3 (9.4%)	0	
No	26 (81.2%)	29 (90.6%)	32 (100%)	
A: Listerine; B: Periogard; C: N&W PRO				

However, mouthwash C was shown to be more preferred by the study participants (*p* = 0.04), as they expressed a significant interest in purchasing the product, unlike the general sentiment towards purchasing mouthwashes A and B.

Another important aspect is that mouthwash C caused significantly less discomfort in the lips, tongue, or throat (*p* < 0.01) and less burning sensation (*p* < 0.001). No irritation of the tongue was observed with any rinsing using mouthwash C (*p* = 0.03), in contrast to the significant irritation reported when using mouthwashes A and B (Fig 5).

**Fig 5 Fig5:**
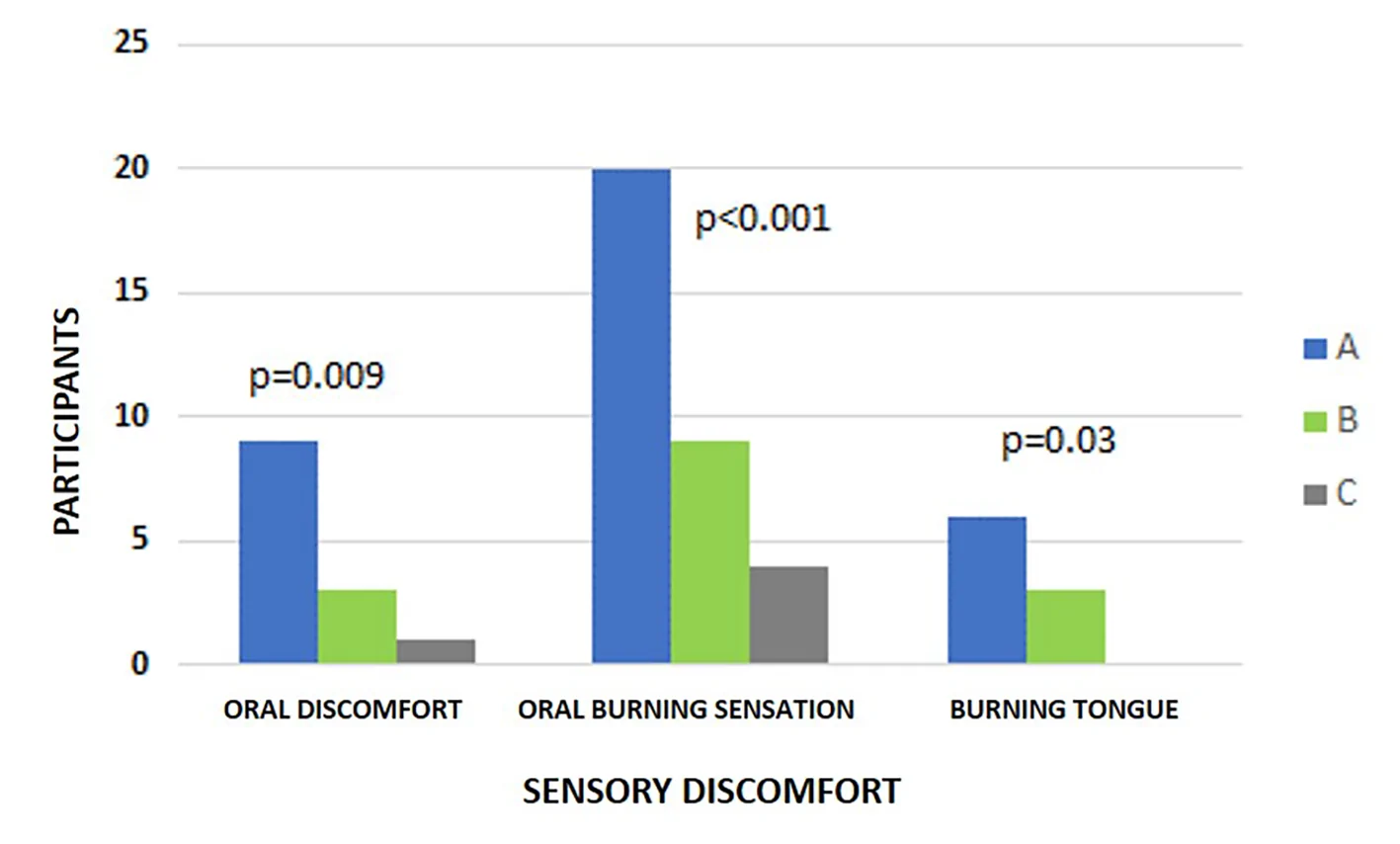
Significantly greater sensory discomfort with mouthwashes A and B compared to mouthwash C. Notably, mouthwash C was the only one that did not cause tongue irritation.

Regarding the idea of developing a product focused on sustainability, the environment, and natural components, 89.6% of the students considered the idea excellent, and 10.4% deemed it good. All participants (100%) expressed support for the inclusion of sustainable practices and natural ingredients in oral hygiene products. Table 1 highlights the main results.

In summary, the primary outcome was that all mouthwashes increased pH effectively, but mouthwash C was preferred for its comfort and lack of irritation. There was also strong support for sustainable and natural products.

## Discussion

The choice of mouthwashes can influence daily sensations in an individual’s oral cavity, affecting sensory comfort by directly impacting taste modifications, burning sensations, and other comorbidities.^5^ In this study, we aimed to evaluate the immediate perception of research participants regarding the use of the main mouthwashes available on the market. The results provided comprehensive insights into the efficacy and acceptance of three different mouthwashes among dental students, as well as the impact of these products on salivary pH and users’ sensory experiences. The main finding highlighted that mouthwash C, based on green tea and hyaluronic acid, caused less discomfort in the lips, tongue, and throat, as well as less burning sensation compared to the essential oil-based (A) and chlorhexidine-based (B) mouthwashes. No tongue irritation was observed with any rinsing involving mouthwash C.

Regarding the sensation of clean teeth, no differences were observed among the studied mouthwashes, indicating that all products provide a satisfactory oral hygiene experience. However, one of the most significant findings of this study was the increase in salivary pH after using all three types of mouthwashes. The initial average pH was 7.0, rising to 8.6 post-use, indicating that all products were effective in increasing salivary pH, which can be beneficial in preventing cavities and periodontal diseases. This increase in saliva pH is crucial as it creates a less acidic environment, unfavourable for the proliferation of cariogenic bacteria and essential for preventing halitosis.^27^


Although the efficacy of the mouthwashes in altering salivary pH was similar, the sensory aspects and acceptance of the products by the students showed significant variations, directly influencing the potential for product acquisition. The green tea and hyaluronic acid-based mouthwash was the only one that did not cause tongue irritation and was preferred by students for possible daily use. This aspect is extremely relevant, as literature reports evident discomfort from using chlorhexidine-based mouthwashes and some essential oils.^2,16,20
^ The absence of irritation and burning with the green tea-based mouthwash may be related to its specific formulation, which includes gentler or more natural compounds, reducing the potential chemical irritants present in other products.

Additionally, the alcoholic content of some mouthwashes, composed of essential oils, can cause a burning sensation in the lips, tongue, and throat. This sensation is more common in individuals with sensitive oral mucosa and may be a limiting factor for daily use.^2^ In our study, supporting previous studies, most participants using essential oil mouthwashes reported a significant burning sensation during use.

The literature reports that essential oils can cause dryness in the mouth for some users due to stimulating the dehydration of oral mucosa. Although rare, allergic reactions to these oils’ components may occur. Symptoms may include skin rashes, itching, and, in more severe cases, swelling of the oral mucosa.^27^ This fact may contraindicate the use of these mouthwashes in people with chronic xerostomia caused by medications or systemic diseases.

Tooth and tongue discolouration is a well-documented side effect of prolonged chlorhexidine use. This discolouration is typically brown or black and occurs due to the interaction of chlorhexidine with chromogenic compounds in the diet and saliva.^24^ Chlorhexidine can cause dysgeusia or taste alteration, leading users to perceive a bitter or metallic taste in their mouths. This alteration may persist during use or for some time after discontinuation. Prolonged chlorhexidine use may also result in oral mucosa irritation, leading to the desquamation of the oral cavity epithelium, causing significant discomfort to the user.^16^ In this study, with the immediate use of chlorhexidine, research participants felt a notable burning sensation in the oral cavity and discomfort in the lips, tongue, and throat, confirming previously described side effects, probably associated with pellicle formation. These side effects reported in the literature emphasise the importance of using mouthwashes based on essential oils and chlorhexidine with moderation and under the guidance of an oral health professional, especially for individuals with pre-existing conditions or oral sensitivity.

Another highlighted aspect was the students’ concern with sustainability and natural ingredients. The positive receptivity from students (100%) towards sustainable products indicates a growing awareness and demand for products that are not only effective but also environmentally conscious. Furthermore, the strong preference for sustainable products suggests that the dental industry should consider incorporating more natural ingredients and environmentally sustainable manufacturing practices. The development of effective products that also meet ecological and patient comfort concerns seems to be a promising path.

A limitation of this study is the sample being comprised only of dentistry students from a single institution, which might not represent the diversity of the general population. Additionally, the evaluation was based on students’ subjective perceptions of taste and comfort, which can vary significantly from person to person. However, it was evident that while all mouthwashes are effective at elevating salivary pH, there are significant differences in user acceptance and the presence of side effects. Green tea-based mouthwashes had better acceptance due to the absence of discomfort and oral irritation, alongside meeting the growing demand for sustainable and natural products.

In addition, based on the research, mouthwashes with ingredients like green tea and hyaluronic acid, which showed higher comfort and fewer side effects, might be particularly beneficial for older patients.^23^ They are often more sensitive to irritation and burning sensations, which were less reported with these formulations. Additionally, raising salivary pH can help prevent cavities and periodontal disease, which are common concerns in older populations. Mouthwashes that prioritise natural and gentle ingredients could promote better adherence and satisfaction among older users.

## Conclusion

This comparative study among essential oil-based, chlorhexidine-based, and green tea/hyaluronic acid-based mouthwashes demonstrated that all mouthwashes were effective in raising pH and providing a sense of oral cavity cleanliness. However, essential oil and chlorhexidine-based mouthwashes produced irritation and discomfort in the tongue, lips, and throat. The green tea/hyaluronic acid-based mouthwash was the only one without reports of tongue irritation.

The clinical relevance of this study lies in its ability to provide insights into the effectiveness and user acceptance of different mouthwashes. By evaluating salivary pH changes and sensory experiences, the study informs dental professionals about formulations that enhance patient comfort and compliance, potentially improving oral health maintenance and prevention of periodontal diseases. In conclusion, green tea and hyaluronic acid mouthwash increases salivary pH with better user comfort and acceptance than chlorhexidine or essential oils.

### Acknowledgements

The authors are indebted to the individuals who enthusiastically participated in the research protocols involved in this study. In addition, the authors thank the whole team from the Implant Dentistry Postgraduation who contributed to the clinical procedures.

## References
